# Histomorphological investigation of intrahepatic connective tissue for surgical anatomy based on modern computer imaging analysis

**DOI:** 10.1002/jhbp.753

**Published:** 2020-07-22

**Authors:** Tetsuo Ikeda, Shinji Okano, Naotaka Hashimoto, Koichi Kimura, Kensuke Kudo, Ryosuke Tsutsumi, Shun Sasaki, Junji Kawasaki, Yu Miyashita, Hiroya Wada

**Affiliations:** ^1^ Department of Integration of Advanced Medicine and Innovative Technology Kyushu University Hospital Fukuoka Japan; ^2^ Department of Endoscopy and Endoscopic Surgery Fukuoka Dental College Fukuoka Japan; ^3^ Department of Pathology Fukuoka Dental College Fukuoka Japan; ^4^ Department of Surgery and Science Graduate School of Medical Sciences Kyushu University Fukuoka Japan

**Keywords:** liver extracellular matrix, computer‐assisted three‐dimensional imaging, AI random forest, proper hepatic capsule, proper hepatic ligament

## Abstract

**Background/Purpose:**

Computer‐assisted tissue imaging and analytical techniques were used to clarify the histomorphological structure of hepatic connective tissue as a practical guide for surgeons.

**Methods:**

Approximately 5000 histological slides were prepared from liver specimens of five autopsied patients. Three‐dimensional (3D) reconstruction was performed and subjected to computer imaging analysis. Scanning electron microscopy was also performed on the liver specimens.

**Results:**

The 3D reconstructed images revealed the running form of the vasculature and the relationship between the hepatic lobule and connective tissue. The hepatic capsule or portal pedicle was consistently located at the periphery of the hepatic lobules. An artificial intelligence random forest approach clearly segmented hepatic cells, type I collagen (CF), type III collagen (RF), and other cells. The hepatic lobule, portal region, and hepatic capsule were significantly distinguished based on CF and RF occupancy. The capsule directly covering the liver lobule with an RF concentration up to 87% was provisionally named the proper hepatic capsule. The existence of a proper hepatic ligament with distinct occupation rates of CF and RF was also suggested.

**Conclusions:**

The identified proper hepatic capsule and ligament can be important markers for demarcating the dissecting layer during surgical procedures.

## Graphical abstract

***Highlight***

Computer‐assisted tissue imaging and analytical techniques were used to clarify the histomorphological structure of hepatic connective tissue. Ikeda and colleagues focused on the capsule surrounding the hepatic lobule itself. The identified proper hepatic capsule and ligament appear to be an important structure for demarcating the dissecting layer during surgical procedures.

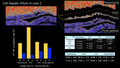

## 1 INTRODUCTION

Anatomical liver resection with portal pedicle isolation is widely approved as an essential procedure owing to its good safety profile and curability potential. Couinaud^1^ proposed the concept of the extrahepatic portal pedicle approach for a left hepatectomy in 1985, although Takasaki et al^2^ were the first to perform the portal pedicle transection method in 1990. Recently, Sugioka et al^3^ proposed anatomical liver resection with portal pedicle isolation based on the liver capsule as the anatomical marker. However, complications such as bleeding and bile duct injury due to the portal pedicle isolation technique are still important problems in anatomical liver resections.

The liver is maintained in an extracellular matrix, and the structure and components of human liver collagens composing the extracellular matrix were comprehensively analyzed in a series of studies performed in the 1980s.^4^, ^5^, ^6^ Despite some important qualitative and quantitative differences, four typical genetic types of collagen are recognized: I, III, IV, and V.^7^ Two methods are commonly used for the solubilization and quantitative analysis of the different genetic types of collagen in the liver, involving pepsin digestion or CNBr cleavage of the remaining connective tissue after removing part of the non‐collagenous proteins.^4^, ^5^, ^6^, ^7^, ^8^, ^9^ Type I collagen corresponds to the thick collagen bundles of the liver and forms the dense connective tissue,^4^, ^5^, ^6^ which is present in the liver capsule, the stroma of the portal tracts and large vessels, around the terminal venule areas, and occasionally inside the liver lobule.^10^, ^11^, ^12^, ^13^ There is a 1:1 ratio of type I to type III collagen.^4^, ^5^, ^6^ Type III collagen corresponds to some but not all of the reticulin fiber (RF) of the liver, which is mixed with type I collagen in the stroma of the large vessels and portal triads and forms a very fine tissue framework in the liver parenchyma.^8^, ^9^, ^10^, ^11^, ^12^, ^13^ Type III collagen has also been found inside the space of Disse with a dose‐dependent association with the processes of Ito cells.^14^, ^15^ Type I collagen fibers stain blue with trichrome stains, pale brown to red with silver stains, and red with picrosirius dye.^16^, ^17^ RF is a small‐diameter fibril that stains black or dark brown with silver stains.^18^ Early studies suggested that collagens I, II, and III could be distinguished based on their different birefringence. However, subsequent studies showed that the color produced is related to the diameter of the fibrils and is not specific to the properties of each genetic type of collagen.^19^

Given the importance of understanding the detailed histomorphological structure of the connective tissue of the liver for surgeons, new approaches are needed to best distinguish individual structures, landmarks, and cell types to properly guide hepatic surgery. To this end, in this study, we examined the structure of the extracellular matrix of the liver using modern computer‐assisted imaging analysis techniques and an artificial intelligence random forest approach. Specifically, three types of image analysis software were used to clarify the structure and morphology of the intra‐hepatic connective tissue. Amira^20^ is an extendable software system for scientific visualization, data analysis, and the presentation of three‐ and four‐dimensional data. It is currently used worldwide by thousands of researchers and engineers in academia and industry owing to its flexible user interface and modular architecture, providing a universal tool for the processing and analysis of data from various modalities. Segmentation of the components in the 3D‐reconstructed images was performed with the trainable Weka segmentation (TWS) machine‐learning tool that leverages a limited number of manual annotations to train a classifier and then segments the remaining data automatically.^21^ The usefulness of the TWS tool was well demonstrated by its utilization in a wide range of imaging pipelines.^21^, ^22^, ^23^ Fiji has become the software of reference for many scientists to meet their image analysis needs, especially in the field of biomedical imaging, including powerful tools to generate sophisticated image processing pipelines and algorithms.^24^

## 2 METHODS

### 2.1 Ethical considerations

This study was approved by the institutional review board of Kyushu University Fukuoka, Japan (permission number: 26‐147).

### 2.2 Tissue sampling

The tissue samples were obtained from five autopsied patients for pathological analysis (three females and two males, 68‐77 years old with a mean age of 74 years) who died of various causes with no apparent liver disorder. The tissue samples were formalin‐fixed and paraffin‐embedded, and a 6‐mm‐thick tissue block was prepared from the hepatic hilum, hepatic venous outflow tract, and parenchyma. In total, 5000 sections were prepared with a thickness of 5 µm each. All of the prepared serial sections were stained with MT.

### 2.3 Three‐dimensional reconstruction

The 3D reconstruction of the serial tissue sections was performed according to a previously described method.^25^ All of the scanned virtual 2D images were then sequentially loaded into the 3D data visualization, analysis, and modeling software Amira 6® (version 6.2; FEI Visualization Sciences Group). Two main aspects of the 3D reconstructed tissues were focused on. First, we evaluated the vasculature running in the hepatic hilum. Approximately 1000 serial images from one case obtained from the hepatic hilum, artery, bile duct, portal vein, and hepatic vein section were processed and reconstructed in 3D. We then evaluated the overall structure of the extracellular matrix in the hepatic hilum. The serial images were aligned, the liver parenchyma was made to be transparent after 3D reconstruction, and the images were processed with built‐in modules such as Align Slice, Voltex, and Arithmetic.

### 2.4 Segmentation and occupation rate of components

**Figure 1:**
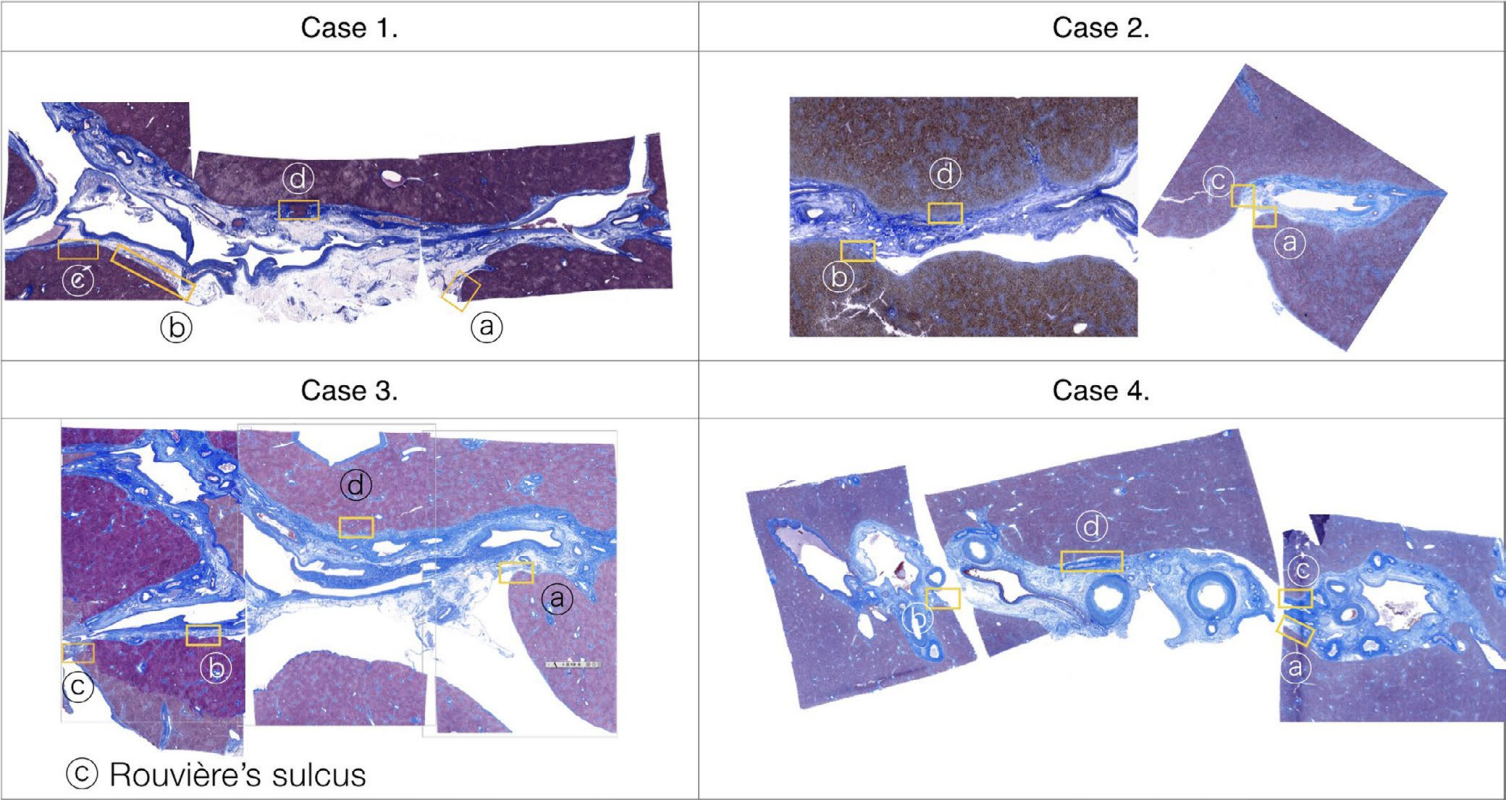
The analysis portions of the four cases. The portions were selected from the left and right corners and from the dorsal side of the hilar portion in each of the cases. Rouvière's sulcus was selected from Case 3 (yellow squares)

The components on each slide were separated using TWS from a high‐resolution slide of 0.22 µm^2^/pixel. From each of the four cases subjected to this procedure, the analysis points were selected from the left and right corners and from the dorsal side of the hilar portion. In the left and right corner of the hepatic hilum, including Rouvière's sulcus, the site at which the hepatic capsule inverts to the portal pedicle was selected as a particularly interesting focal point. Four portions from each of the four cases were selected (Figure 1).

**Figure 2:**
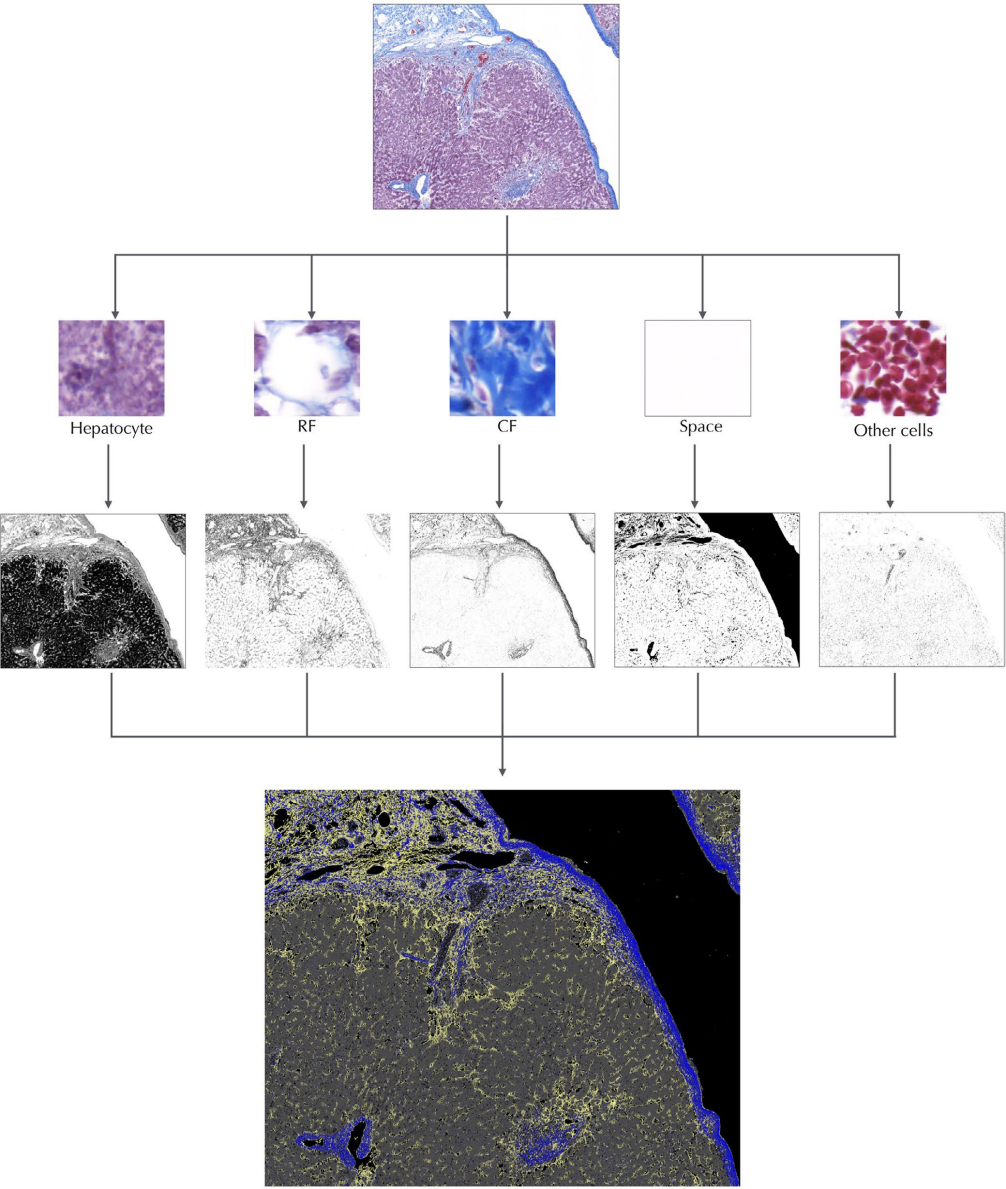
The segmentation procedure using trainable Weka segmentation. From the portion selected from each slide, hepatocytes, CF, RF, space, and other cells, such as red blood cells and lymphocytes, were identified as components. A specific color was assigned to each of the five components and overlaid onto a single image

Identification and segmentation of the different components on slides were conducted with the TWS plugin of ImageJ (https://imagej.net/Trainable_Weka_Segmentation.^21^ At this stage, a pixel‐level classifier was trained for distinguishing hepatocytes, type I collagen (collagen fiber, CF) depicted as thick collagen bundles, and type III collagen depicted as large quantities of reticulin fiber (RF). Other cells, such as red blood cells and lymphocytes, and spaces were identified as liver components with the help of a skilled cytopathologist (Figure 2). This segmentation was achieved by drawing lines/selection through the areas of interest and assigning them to a specific class. The occupancy rate of the five components (hepatocyte, CF, RF, other cells and space) inside each ROI was measured with the Fiji tool of ImageJ (version 2.00‐rc‐68/1.52f; National Institutes of Health).

### 2.5 Specimen preparation for scanning electron microscopy

To determine the specific forms of the collagen fibers at the hepatic hilum, scanning electron microscopy (SEM) was conducted as previously described.^26^, ^27^ In brief, the prepared specimens were placed on a sample table (diameter: 15 mm), coated with osmium, stored in a desiccator, and examined using a Hitachi SU 8000 ultra‐high‐resolution field‐emission scanning electron microscope at Kyushu University's Center for Advanced Instrumental Analysis.

### 2.6 Statistical analysis

JMP Pro software, version 13 (SAS Institute Inc.) was used for all statistical analyses. To evaluate the statistical significance of the results, including the differences in the occupancy rates of each component between cases and structures, Student's equal variance t‐test and Wilcoxon unequal distribution nonparametric tests were performed.

## 3 RESULTS

### 3.1 The main findings of the 3D reconstructed serial sections

Animation S1 shows the structure of the vasculature in the hepatic hilum. The right portal pedicle, including the portal vein, hepatic artery, and bile duct, observed in this image was reconstructed from approximately 600 serial sections of Case 4. This reconstruction demonstrates that the portal vein runs in the central portal pedicle, and the hepatic artery with branches and bile duct run around the portal pedicle. The hepatic artery and bile duct run across the portal vein near the hepatic hilum.

Animation S2 demonstrates the 3D evaluation of the overall structure of the extracellular matrix in the hepatic hilum, which was reconstructed from about 1200 serial sections from Case 3. When the hepatic parenchyma was removed by image processing, it became clear that the hepatic lobules were held by the hepatic capsule in the outer wall and in the inner wall by the portal pedicle sheath. The hepatic capsule and portal pedicle sheath are in the same position to maintain the morphology of the liver. Animation S3 was reconstructed with 20 serial sections of Case 3 within Rouvière's sulcus, which is the portal pedicle sheath exposed on the liver surface. The hepatic capsule or portal pedicle was located at the periphery of the hepatic lobules as shown by the line encompassing the hepatic lobule around the central vein. Animation S4 was reconstructed with 100 serial sections from the hepatic hilum of Case 3. When the hepatic parenchyma was removed by image processing, it became clear that the portal pedicle at the hepatic hilum was intertwined with fine silky fibers. Although these fine silky fibers were also observed in Case 3, they were denser in the sections from Case 2. Animation S5 was reconstructed from only 10 serial sections from Case 1. The density of the fine silky fibers was even less than that of Case 2.

### 3.2 Segmentation and occupation rates of components on the slides

**Figure 3:**
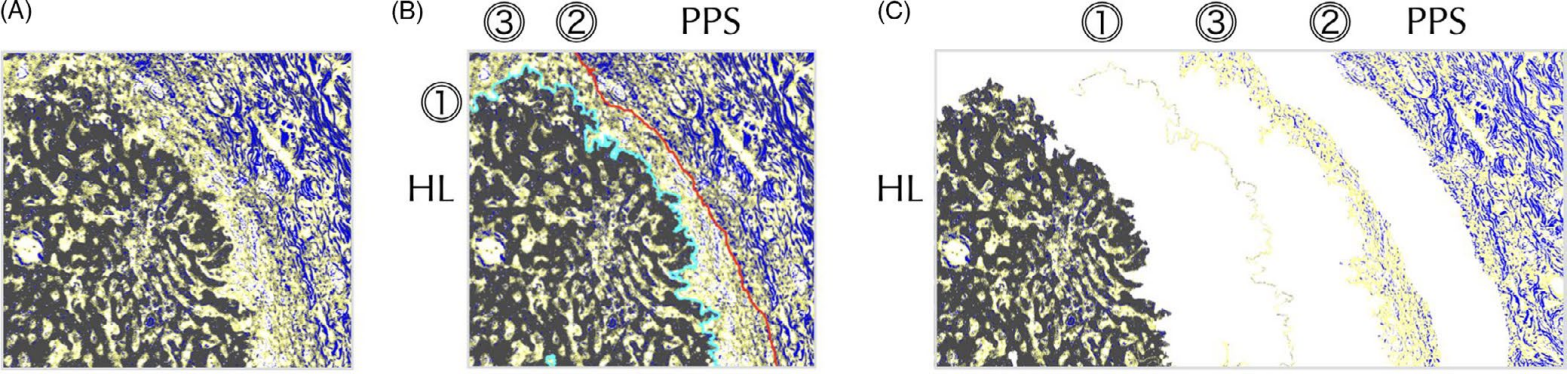
The methods of segmentation for PHC and PHL were independent regions. A, shows image segmented into five components for case 3. The five segmented components are labeled according to color: CF is blue, RF is yellow, hepatocytes are gray, and other cells and spaces are white. B, shows the method of identification of PHC and PHL. A cyan line was drawn along the outer edge of the hepatic lobules and a red line was drawn along the outer edge of the portal pedicle sheath. C, shows segmented and line ⓵ separation as a PHC and band ⓷ between the cyan line ⓵ and red line ⓶ as a PHL. HL, hepatic lobule; PPS, portal pedicle sheath; ⓵, a cyan line is drawn along the outer edge of the hepatic lobule as a PHC; ⓶, a red line is drawn along the outer edge of the portal pedicle sheath; ⓷, the band between cyan line ⓵ and red line ⓶ as a PHL

**Figure 4:**
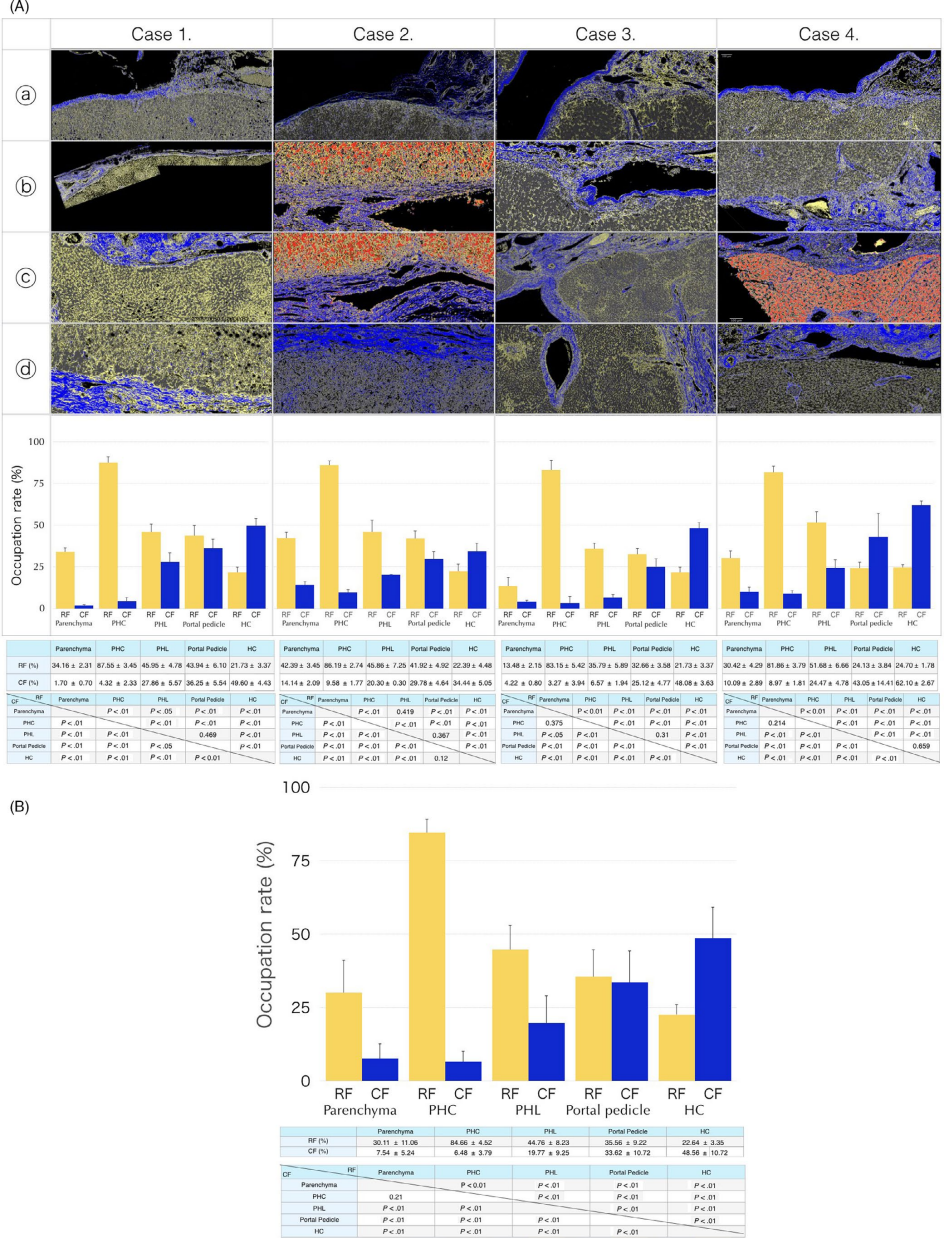
The images segmented components from each portion extracted from each case. The graphs show the CF and RF occupancy rates in the parenchyma, PHC, PHL, portal pedicle sheath, and hepatic capsule for each portion. The ratio of the CF and RF was constant within each region, and there was a significant difference in the ratio between regions in each case (A). Although there was between‐case variation in the ratio of CF and RF in the portal pedicle, a collective significant difference in the comparison of the ratio of CF and RF in the four cases was also recognized (B). The RF and CF occupation rates for each region are shown in the upper table. The difference in occupation rate in the comparison of RF and CF between regions is shown in the bottom tables. CF, Type I collagen fiber; HC, hepatic capsule; PHC, proper hepatic capsule; PHL, proper hepatic ligament; RF, reticular fiber. Data are expressed as the means and standard deviation. *P* < .01 (Student's *t*‐test of equal variance)

The hepatic cells, CF, and RF were clearly segmented with the Weka segmentation tool. The five segmented components were labeled according to color: CF is blue, RF is yellow, hepatocytes are gray or red, and other cells and spaces are black or white. A line was drawn along the outer edge of the hepatic lobules (cyan line in Figure 3B and Animation S6), which corresponds to the outer edge of the hepatocytes that constitutes the limiting plate. Another line (the red line in Figure 3B and Animation S6) was drawn along the outer edge of the portal pedicle sheath. The band between the cyan line ⓵ and red line ⓶ was segmented. Line ⓵ and band ⓷ were added to the parenchyma, portal pedicle, and hepatic capsule as independent regions (Figure 3B,C). The regions of interest (ROIs) were rectangular with a length of 2000‐4000 pixels and a width of 500 pixels between the liver parenchyma region and portal pedicle region. Five to ten ROIs were extracted from one selected portion. The occupied ratio of each of the five components (hepatocytes, CF, RF, other cells, and space) was measured at four regions (line ⓵, band ⓷, parenchyma and portal pedicle sheath or hepatic capsule), respectively, in the ROI of four selected portions (right hilum, left hilum, hepatic plate, and Rouvière's sulcus) for each of the four cases. As shown in Figure 4, the occupied ratio of RF on line ⓵ was on average, nearly 85% for the four cases, which was significantly greater than that of the other regions. In addition, band ⓷ had significantly different occupation rates for RF and CF compared with the parenchyma, portal pedicle, and hepatic capsule. Based on this finding, line ⓵ was provisionally named a “proper hepatic capsule (PHC),” and band ⓷ was named a “proper hepatic ligament” (PHL). The ratio of the CF and RF was constant in each region, and there were significant differences in the ratio between regions in each case (Figure 4A). Although there was a variation between cases in the ratio of CF and RF in the portal pedicle sheath and hepatic capsule, a significant difference in the collective ratio of CF and RF in the four cases was observed (Figure 4B). The positional relationship among the hepatic lobule, PHC, PHL, and portal pedicle or hepatic capsule are shown in Animation S7. The PHC directly covers the liver lobule, and PHL covers PHC and connects to the portal pedicle sheath. The contact state of PHC, PHL, and the portal pedicle sheath and thickness of PHL are shown in Animation [Link], [Link], [Link], [Link], [Link], [Link]. In Cases 1 and 4, PHC (cyan line) is in contact with the portal pedicle sheath across PHL, which has only a window frame structure in a row (Animations S8.1 and S8.2). Alternatively, in Case 2, a thick PHL mesh was observed between the PHC and portal pedicle sheath (Animations S8.3 and S8.4). In Case 3, a moderately thick PHL was observed between the PHC (cyan line) and portal pedicle sheath (Animations S8.5 and S8.6).

**Figure 5:**
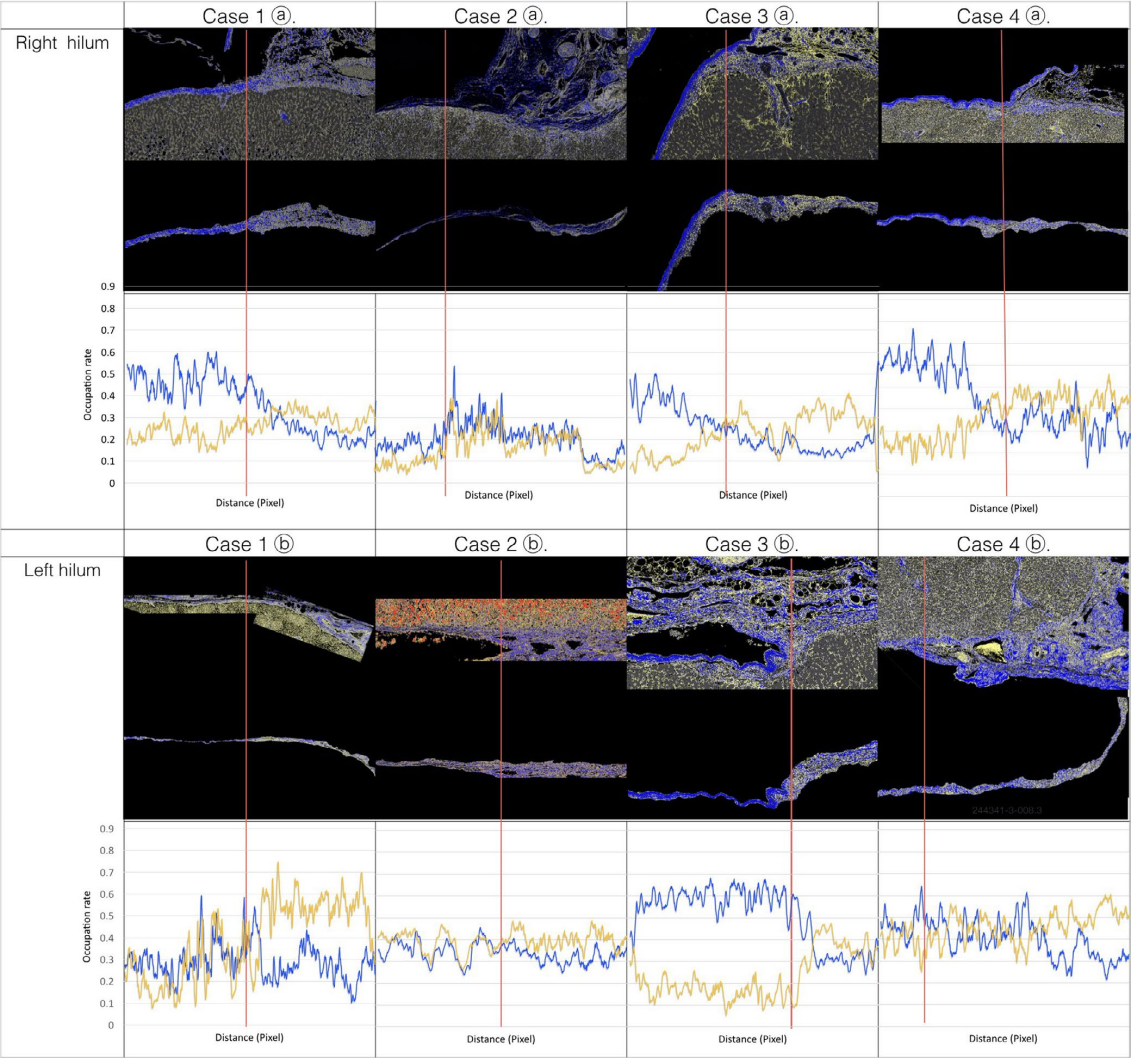
The images segmented components from portions extracted from the right or left hilum in each case. The upper images are the selected portions. In the left and right hepatic hilum, the upper row is images segments of the whole portion, the lower row is the ROIs extracted from the parts directly covering the hepatic lobules that are the targets of the RF and CF occupation rate measurement. The graphs show the ratio of CF and RF measured from the hepatic capsule to the portal pedicle sheath. At the turning point (red line) from the hepatic capsule to the portal pedicle, there was a tendency for the CF occupancy to decrease and for the RF occupancy to increase

Figure 5 shows the ratio of CF and RF measured in the direction from the hepatic capsule to the portal pedicle sheath where the hepatic capsule turns toward the portal pedicle sheath in both the right and left hepatic hilum. The hepatic capsule comprised approximately 50% CF, except in Case 2. At the turning point from the hepatic capsule to the portal pedicle sheath (red line), there was a tendency for the CF occupancy ratio to decrease and the RF occupancy ratio to increase.

As shown in Animation S9, the main part of the hepatic capsule is inverted to the extra‐hepatic portal pedicle sheath, and the remaining part is transferred to the intra‐hepatic portal pedicle sheath. This finding is more clearly observed in cases with a high CF occupation ratio of the hepatic capsule (Cases 1, 3, and 4).

### 3.3 SEM findings

**Figure 6:**
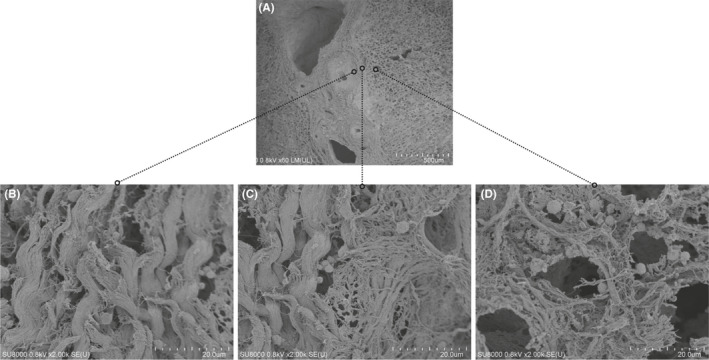
The SEM findings of the right hepatic hilum in case 5. A, shows at low magnification (×60). B‐D, are at high magnification (×2000). At the site of the portal pedicle sheath (B), a thick and dense bundle of collagen fibers was lined up in a certain direction, whereas thin, low‐density fine fibers formed a lattice frame surrounding the melted hepatic cell cord at the site of the parenchyma (D). At the site between the parenchyma and portal pedicle (C), thin bundles of reticulin fibers were entangled with the thick collagen fibers

Figure 6 shows SEM images of the portal pedicle and parenchyma excised at the right hilum from Case 5. This specimen was soaked in 8% NaOH and repeatedly rinsed with distilled water to extract only collagen tissue (containing CF and RF); thus, almost all of the hepatocytes and other cells were removed. SEM imaging of the right hepatic hilum was first performed at low magnification (×60) (Figure 6A), and then detailed examinations of three sites were performed at high magnification (×2000) (Figure 6B‐D). At the site of the portal pedicle (Figure 6B), thick and dense bundles of fine collagen fibers were oriented, whereas thin, low‐density fine fibers formed a lattice frame surrounding the melted hepatic cell cord at the site of the parenchyma (Figure 6D). At the site between the parenchyma and portal pedicle (Figure 6C), low‐density bundles of RF were entangled with thick, dense collagen fibers. This layer corresponds to the site of the PHC and PHL observed in the optical microscope findings.

## 4 DISCUSSION

Histomorphological investigation using modern computer image analysis has three distinct advantages compared with traditional approaches. The first advantage is that evaluations can be made for multiple cross‐sections, such as the XY plane and ZY plane, in addition to the XZ plane used for normal histological diagnosis. As a result, it was possible to obtain a better understanding of the 3D structure of the extracellular matrix of the liver, clearly showing the links between the connective tissues. In addition, it is possible to visualize the detailed running form of the vessels in the portal pedicle. The second advantage is that the artificial intelligence random forest approach using TWS allows automatic analysis of a large amount of data, enabling the segmentation of tissue sections using only MT staining. Type I CF is typically stained brown, and type III RF is stained black using a silver impregnation method. Therefore, we also tried to classify these components by silver staining, but only a small part could be distinguished and was not clearly identified. The third advantage is that the degree of staining can be converted into a numerical value of the total number of pixels, or the intensity of staining in one pixel can be displayed to a maximum of 255 levels. This feature allows for conducting quantitative analysis of the different types of collagen in the liver without requiring more complex solubilization and quantitation methods.

Based on this approach, it was possible to distinguish each region of the liver, such as the parenchyma, portal pedicle, hepatic capsule, PHC, and PHL, according to the difference in the occupied ratio of RF and CF. Approximately 38% of the liver parenchyma is composed of connective tissue, which is mainly RF, and the hepatic lobule is completely covered with PHC that is composed of approximately 85% RF. The width was variable in each case, but the PHC was generally covered with PHL comprising 45% RF and 20% CF. The PHL is in contact with the portal region or the hepatic capsule. Although there was variability among the cases, the portal pedicle sheath was equally composed of about 34% CF and RF in the average of four cases, but the occupation rate of CF in the hepatic capsule was the highest at approximately 50%. The results regarding the occupation ratio of CF and RF in each region are consistent with previous studies using solubilization and quantitation methods.^28^

An important finding of this study is the identification of PHC mainly composed of RF that surrounds the hepatic lobule. The ring of hepatocytes abutting the connective tissue of the portal region is called the periportal limiting plate. The RF surrounding the hepatocytes constituting the limiting plate forms a capsule. This capsule bundles hepatocyte cell cords, covers the hepatic lobule and abuts the connective tissue of the portal region separated by the Mall space, which is a lymphatic cavity. This capsule was designated as the proper hepatic capsule. Although the thickness of the PHC was only 3‐20 pixels (0.066‐0.44 µm), it nevertheless thicker than the surface layer of the hepatic capsule, which is composed of a monolayer of the mesothelium.

Moreover, the PHL was also defined in this study, with an average occupied ratio of RF and CF of 44.76% and 19.77% (Table in Figure 4B), respectively, for four cases. Therefore, the PHL has a lower RF and higher CF composition than the parenchyma, but has a higher RF and lower CF composition than the portal pedicle or hepatic capsule. A thicker PHL was present at the boundary between the hepatic lobules and portal pedicle sheath or hepatic capsule in cases with marked liver fibrosis. Fibrosis at the margin of the hepatic lobule was particularly conspicuous in the case with obvious fibrosis of the liver parenchyma. The PHL is a structure in which collagen fibers have invaded from the portal region into the lattice‐like or mesh‐like RF that originally surrounded the lost hepatocytes. This inference is further supported by the fact that the PHL of Case 2 with marked fibrosis was thick and mesh‐like, whereas the PHL was the only lattice surrounding the Mall space in Cases 1 and 4 where the hepatocytes composing the limiting plate were lined up. Case 3, with moderate fibrosis, had a moderately thick PHL (Animations S7 and S8).

In Case 4, a part at which the portal pedicle peeled off the liver parenchyma was observed at a thickness of about 400 µm on the hepatic plate, which was probably due to the liver removal procedure. Further detailed observation of this peeling point showed that the hepatic lobule remained encased in the PHC, and the peeling line was equivalent to the PHL (Animation S10).

The simulated procedure for adapting the findings of this study to portal pedicle isolation is as follows. First, cut the hepatic capsule until the PHL is reached. Then, grasp the portal pedicle sheath and pull while the hepatic parenchyma is pushed in the opposite direction and separate the parenchyma from the portal pedicle (Animation S11).

The hepatology textbook^29^ describes the relationship between the hepatic capsule and intra‐hepatic and extra‐hepatic portal pedicle sheaths as follows: the connective tissue that constitutes the hepatic capsule wraps around the portal vein, hepatic artery, bile duct, lymphatics, and nerves that enter and exit the liver from the hilar part, and then enters the liver where it is distributed as a skeleton in the parenchyma. In this study, we found that most of the hepatic capsule mainly composed of CF is inverted to the extra‐hepatic portal pedicle sheath at the site where the portal pedicle penetrates the liver. This relationship could be supported by the fact that the occupancy ratio of CF in the hepatic capsule dropped from about 50% to about 30% at the site where the portal pedicle penetrates the liver, which is the same ratio found in the portal pedicle sheath.

In British and American books, periportal connective tissue in the liver is described as portal area or portal tract. In this study, the part of the peripheral connective tissue that touches the liver parenchyma was described as the portal pedicle sheath.

The cases from death due to causes other than liver disease were examined. Cases with liver cirrhosis, hepatocellular carcinoma, and chronic hepatitis that may affect the limiting plate should be investigated. In addition to the hepatic hilum, the structure of the extracellular matrix around the hepatic vein, and the relationship between the hepatic capsule and hepatic vein wall should be clarified next.

In conclusion, histomorphological investigation of the intrahepatic connective tissue using computer‐assisted tissue imaging and analytical techniques was a remarkably useful method for gaining a better anatomical understanding that can guide the surgery. In this study, we focused on the capsule surrounding the hepatic lobule itself and inferred the existence of a PHC and PHL. Although more cases will need to be considered to validate these findings, the PHC and PHL appear to be an important structure for demarcating the dissecting layer in surgical procedures.

## CONFLICT OF INTEREST

Authors declare no Conflict of Interest for this article.

## Supporting information

AnimationS1

AnimationS2

AnimationS3

AnimationS4

AnimationS5

AnimationS6

AnimationS7

AnimationS8.1

AnimationS8.2

AnimationS8.3

AnimationS8.4

AnimationS8.5

AnimationS8.6

AnimationS9

AnimationS10

AnimationS11
